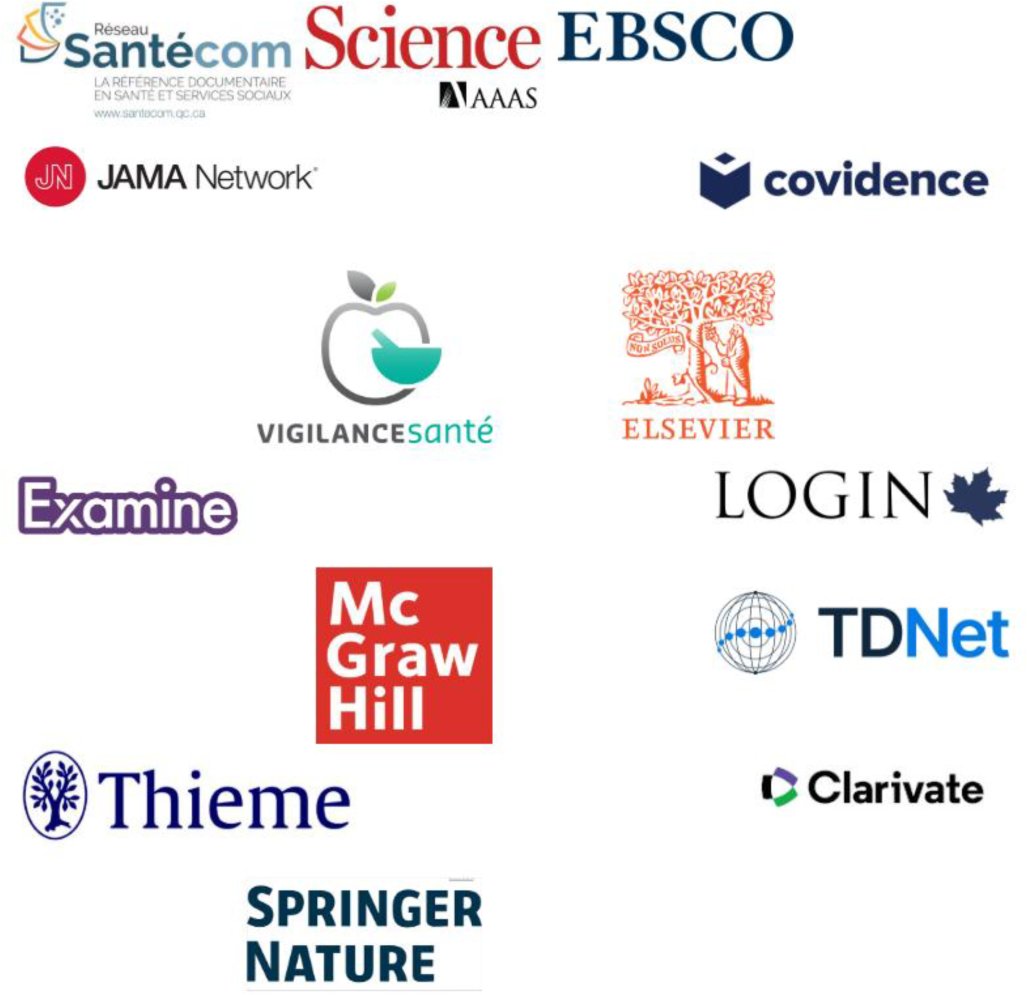# SALUTE TO SPONSORS

**Published:** 2026-08-03

Thank you to the 2026 conference sponsors! Your generous and steadfast support for the CHLA/ABSC is appreciated.

## PLATINUM SPONSORS


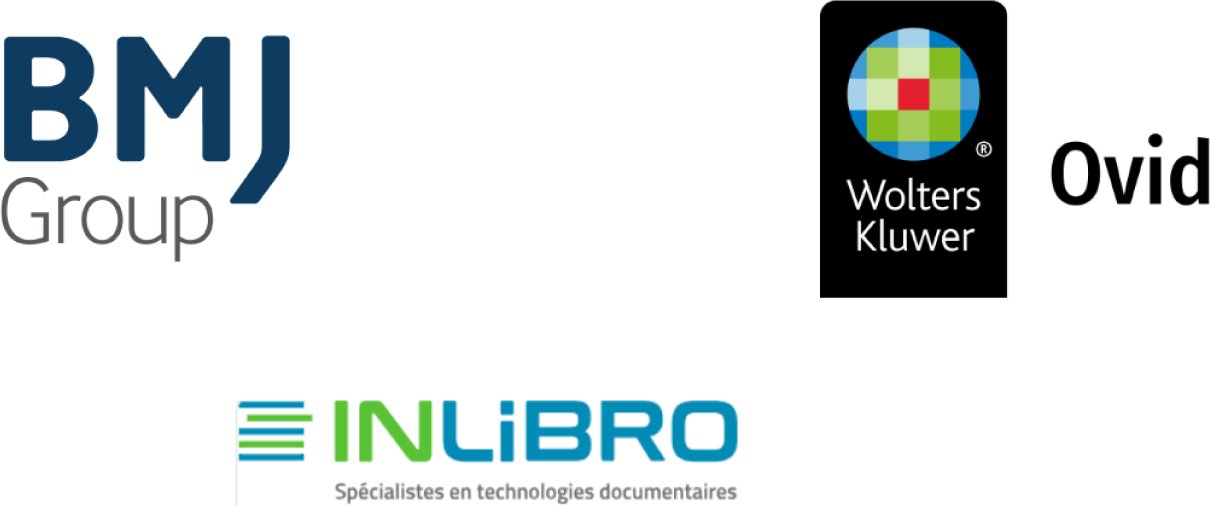


## GOLD SPONSORS


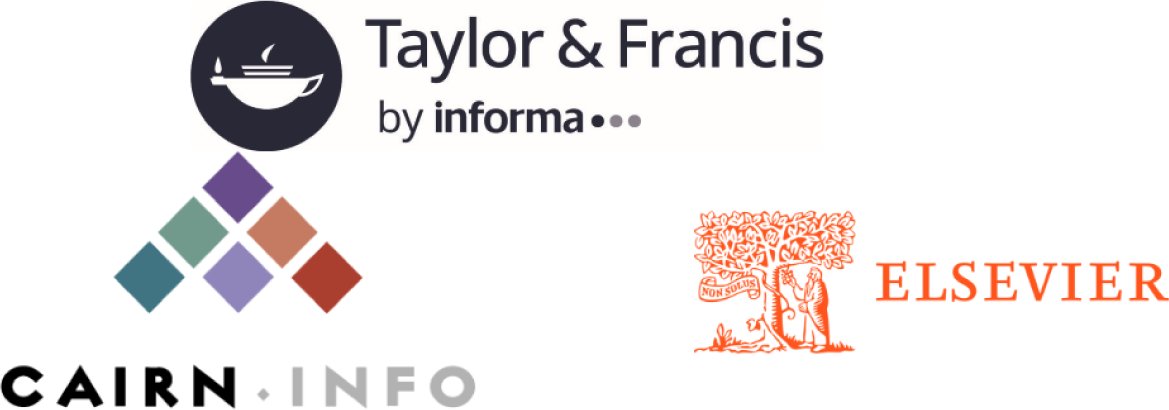


## SILVER SPONSORS